# Computational identification of clonal cells in single-cell CRISPR screens

**DOI:** 10.1186/s12864-022-08359-1

**Published:** 2022-02-15

**Authors:** Yihan Wang, Shiqi Xie, Daniel Armendariz, Gary C. Hon

**Affiliations:** 1 Cecil H. and Ida Green Center for Reproductive Biology Sciences, University of Texas Southwestern Medical Center, Dallas, TX 75390 USA; 2 Department of Bioinformatics, Department of Obstetrics and Gynecology, University of Texas Southwestern Medical Center, Dallas, TX 75390 USA

**Keywords:** Single-cell CRISPR screens, Single-cell genomics, CRISPR

## Abstract

**Background:**

Single-cell CRISPR screens are powerful tools to understand genome function by linking genetic perturbations to transcriptome-wide phenotypes. However, since few cells can be affordably sequenced in these screens, biased sampling of cells could affect data interpretation. One potential source of biased sampling is clonal cell expansion.

**Results:**

Here, we identify clonal cells in single cell screens using multiplexed sgRNAs as barcodes. We find that the cells in each clone share transcriptional similarities and bear segmental copy number changes. These analyses suggest that clones are genetically distinct. Finally, we show that the transcriptional similarities of clonally expanded cells contribute to false positives in single-cell CRISPR screens.

**Conclusions:**

Experimental conditions that reduce clonal expansion or computational filtering of clonal cells will improve the reliability of single-cell CRISPR screens.

**Supplementary Information:**

The online version contains supplementary material available at 10.1186/s12864-022-08359-1.

## Background

CRISPR screens are powerful genetic tools to study the function of genes and regulatory elements genome-wide. Traditional CRISPR screens rely on a phenotypic selection step such as proliferation. Recently, single-cell CRISPR screens have been developed to link genetic perturbations with high content transcriptome-wide phenotypes [1–5]. However, standard procedures for single-cell CRISPR screens have not been systematically evaluated, and how certain experimental parameters affect data interpretation remain understudied. One such parameter is clonal cell expansion. Cancer cells are heterogeneous, and distinct clones have genetic features that facilitate proliferation [6, 7]. Excessive clonal cell expansion could potentially bias cell-based screening by oversampling highly proliferative clones, thereby increasing false signals or decreasing true signals.

Traditional bulk CRISPR screens analyze millions of cells, which limits clonal expansion artifacts [8]. However, in single-cell CRISPR screens, a relatively smaller number of cells can be affordably sequenced, which increases the risk of clonal expansion. Quantification of clonal cells is necessary to assess the clonality in single cell screens.

Single-cell CRISPR screens have introduced multiple sgRNAs per cell to increase throughput [4, 5, 9]. We reason that the combination of sgRNAs in a cell can serve as a barcode to track clonal cells. We developed a computational strategy to identify clones based on multiplexed sgRNA barcodes in sequenced cells. To test this approach, we performed a single-cell screen in breast cancer cells. We identified distinct populations of clonal cells, and we show that removal of clonal cells significantly reduces false discovery. Finally, we identify segmental copy number changes by comparing the transcriptomes of clonal cells, and these data suggest that clones are also genetically distinct. In sum, this approach can be used to improve the quality and interpretation of single-cell CRISPR screens, and it can allow clonal lineage information [10–13] to be derived from these data.

## Results

### Multiplexed sgRNAs serve as a clonal barcode

**Fig. 1:**
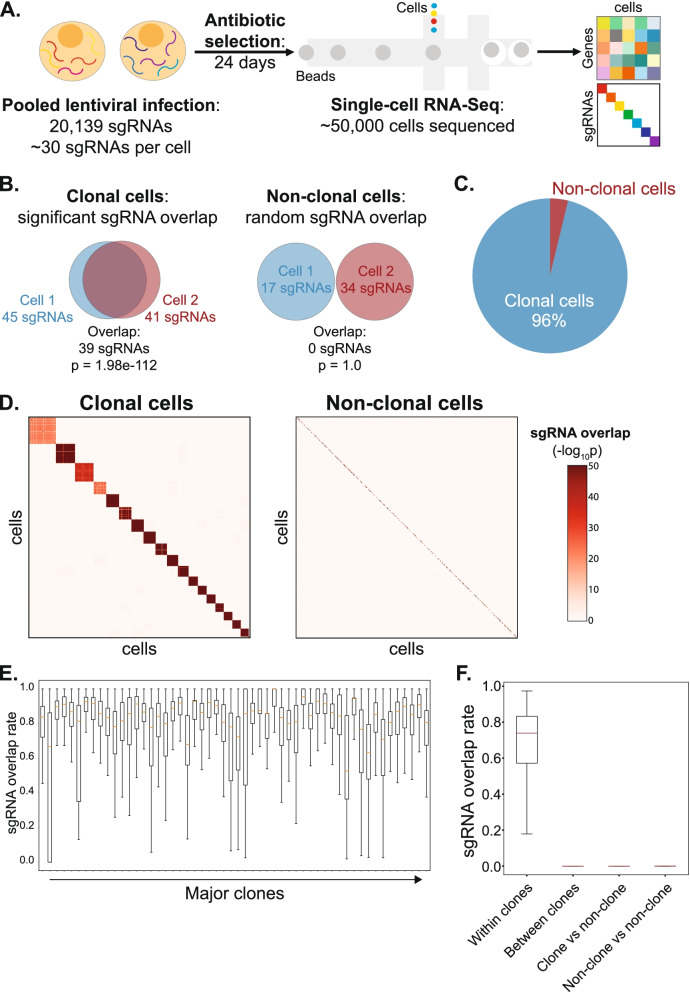
Multiplexed sgRNAs serve as a clonal barcode. **A**. Overview of single-cell CRISPR screen performed in this study (~ 20 k sgRNAs, ~ 50 k cells sequenced, ~ 30 sgRNAs per cell). **B**. Examples of clonal (left) and non-clonal (right) cells. Shown is the overlap of sgRNAs between two sequenced cells and the significance of this overlap (hypergeometric *p*-value). **C**. The proportion of clonal cells and non-clonal cells in the single-cell CRISPR screen dataset. 96% of sequenced cells have some degree of clonality, with at least one other cell sharing significant overlap of sgRNAs. **D**. We sampled 500 random cells from either clonal (left) or non-clonal (right) populations and then calculated *p*-values of sgRNA overlap for each pair of sampled cells. Shown is a heatmap of these *p*-values. **E**. Distribution of pairwise sgRNA overlap rate in 54 major clones. Given two cells, the sgRNA overlap rate is the number of shared sgRNAs (intersection) divided by the number of total sgRNAs (union). **F**. Pairwise sgRNA overlap rate for cells: in the same clone, between different clones, between clones and non-clones, and within non-clonal cells. Given two cells, the sgRNA overlap rate is the number of shared sgRNAs (intersection) divided by the number of total sgRNAs (union)

We reasoned that the presence of multiple sgRNAs in a cell could be used to barcode distinct clones. Therefore, we infected MDA-MB-231 cells stably expressing the CRISPRi effector dCas9-KRAB with a high MOI virus spanning a complex library of 20,000 distinct sgRNAs (Fig. 1A). To enhance our ability to identify clones, we performed these analyses in low cell numbers (0.2 million cells) with long antibiotic selection (24 days). Overall, we performed single-cell RNA-Seq on ~ 55,000 cells passing robust data quality standards including the removal of dying cells and cell doublets by cell hashing [14] (Supp. Fig. 1, see Methods). On average, we detected 33.9 sgRNAs (median 32) expressed in each cell and the average UMI for each detected sgRNA in a cell is 15.1 (Supp. Fig. 1).

To identify clones, we developed a computational strategy based on the hypergeometric test to group clonal cells using the set of sgRNAs expressed in each cell as its clonal barcode (Fig. 1B). Overall, we identified 3541 clones spanning 96% of all sequenced cells (Fig. 1C). On average, each clone contained 14 cells. We identified 54 major clones each with more than 100 cells (Supp. Fig. 2) and 1886 high-confidence non-clonal cells. This analysis was robust to the approach used to identify sgRNAs in each cell (Supp. Fig. 3).

To assess the accuracy of our clone definitions, we compared sgRNAs across clones (Supp. Fig. 2). If each clone is distinct, then we expect sgRNAs to be shared within each clone but not between clones. We therefore calculated the *p*-value of sgRNA overlap between pairs of cells in clonal and non-clonal groups (Fig. 1D). We observe that cells from the same clone exhibit statistically significant overlap of sgRNAs, while cells from different clones do not. A rare set of cells exhibiting sgRNA overlap with multiple clones coincided with cell hashing doublets, suggesting that sgRNAs can also identify cell doublets [14].

To quantify these observations, we compared the sgRNA overlap rate within and between clones. On average, cells in each major clone share 77.3% of sgRNAs (Fig. 1E). In contrast, cells between major clones exhibit significantly less overlap (0.068%) (Fig. 1F). Similarly, clonal cells do not exhibit significant sgRNA overlap with non-clonal cells (0.036%), and likewise non-clonal cells rarely share sgRNAs (0.35%).

Next, we applied our computational pipeline to two publicly available datasets [4, 5]. We rarely found clonal expansion in these datasets: 99% (K562), 98% (K562), and 83% (HeLa) of sequenced cells are high-confident non-clonal (Supp. Fig. 4). Notably, these studies used more cells and shorter antibiotic selection time, which reduced clonality. Overall, these results suggest that our approach can identify clonal cells using multiple sgRNAs as a barcode.

### Clonal cells identified by sgRNA barcodes have distinct genomic features

**Fig. 2:**
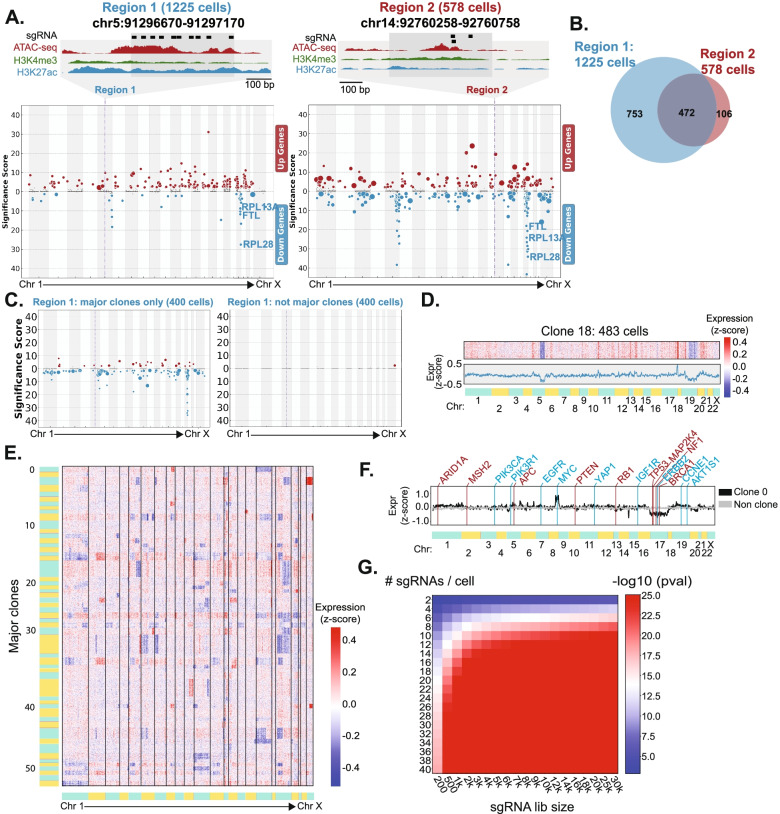
Perturbation clones have distinct genomic features. **A**. Manhattan plots showing the differentially expressed genes for cells with sgRNAs from two independent genomic regions. X-axis: genes ordered by chromosomal coordinate. Y-axis: significance differential gene expression. Positive values: up-regulated genes; negative values: down-regulated genes. Note that these distinct perturbations share the same sets of differential expressed genes in chromosome 5 and chromosome 19. **B**. We sequenced 1225 and 578 cells with sgRNAs targeting chr5:91296670–91,297,170 and chr14:92760258–92,760,758, respectively. Shown is the overlap of cells, which is statistically significant (*p* = 3.42e-35, hypergeometric *p*-value). **C**. We randomly selected 400 cells from either major clonal cells or other cells. The Manhattan plots show that the signals come from clonal cells. **D**. Clone 18 represents most of the overlapping cells in 2**B**. (top) For all 483 cells in clone 18, the heatmap shows the z-score normalized expression of all genes ordered by chromosomal coordinate. (bottom) Average values across all cells. The putative segmental deletions of chromosomes 5 and 19 are consistent with the differentially expressed genes in Fig. 2**A**. **E**. For each of the 54 major clones identified, the heatmap shows the z-score normalized expression of all genes ordered by chromosomal coordinate. **F**. For Clone 0, shown is the average z-score normalized expression of genes, ordered by chromosomal coordinate. Several tumor suppressors (red) and oncogenes (red) that overlap potential regions of segmental amplification or deletion, respectively, are labeled. **G**. Power analysis indicates the p-value of sgRNA overlap in two sequenced cells as a function of sgRNA library size and the number of sgRNAs detected per cell. We assume that clonal cells share 75% of detected sgRNAs

To understand the impact of clones on single-cell CRISPR screens, we performed differential expression analysis of targeted genomic regions. Unexpectedly, we observed that some perturbations targeting independent genomic regions often share many differentially expressed genes (Fig. 2A). For example, two perturbed regions (chr5:91296670–91,297,170 and chr14:92760258–92,760,758) are on different chromosomes but share clustered down-regulation of multiple genes on chromosome 19 (RPL28, RPL13A, FTL). Interestingly, we noticed that a large proportion (35%) of these cells share sgRNAs from both perturbed regions (Fig. 2B). This overlap is statistically unlikely to occur by chance (*p* = 3.42e-35, hypergeometric), suggesting that overlapping cells are clones. Indeed, we found that 98% of the overlapping cells come from major clone 18.

To assess whether clonal cells can influence the identification of differentially expressed genes, we repeated our analysis in clonal and non-clonal cells. We find that clonal cells retain clusters of differential gene expression, especially on chr19, while non-clonal cells do not (Fig. 2C). Simulations also confirm that increasing numbers of clonal cells increases the detection of clone-specific hits (Supp. Fig. 5). These results suggest that removal of clones is necessary to avoid false discovery. As an alternative approach, recently developed computational frameworks for single-cell screens such as SCEPTRE and Normalisr could be used to adjust for clones by modeling them as covariates [15, 16].

Since clone 18 has clustered down-regulation of genes in chromosome 19, we hypothesized that the cells in this clone may have a segmental deletion of chromosome 19. To test the hypothesis, we used the grouped transcriptomes to visualize relative changes in genomic copy number. We first calculated expression z-scores for each cell and gene pair. Our rationale is that clusters of genes with positive z-scores indicate gain in copy number while clusters of negative z-scores indicate copy number loss. We removed genes expressed in less than 10% of cells to focus on genes with the most robust signal, which are 8282 genes in total. Our analysis shows that clone 18 exhibits clustered depletion of gene expression on both chromosomes 5 and 19 (Fig. 2D**)**, consistent with the clustered down-regulated genes observed in differential expression analysis (Fig. 2A). Thus, these results suggest that cells from clone 18 may have segmental deletions of these chromosomes.

Next, we expanded this analysis across the 54 major clones. We found evidence for distinct copy number gains and losses in different clones (Fig. 2E). For example, Clone 45 exhibits copy number loss across chromosome 17. In contrast, Clone 31 exhibits copy number loss across multiple chromosomes (chr2, chr6, chr7, chr19, chr21). Interestingly, segmental deletion of chromosome 19 frequently occurs in many clones (18 clones, 3625 cells). In addition, we find that the signatures of structural changes in clonal cells are distinct from those in non-clonal cells (Supp. Fig. 6). As an alternative approach, we also visualized transcriptomes with t-SNE analysis. While the cells from the same major clone often did not fall into a single cluster, clonal cells do have lower Shannon entropy compared with non-clonal cells (Supp. Fig. 7) [17], indicating that clonal cells share similar transcriptomes. Overall, these results suggest that clones identified from sgRNA barcodes are also genetic clones with distinct genomic structure. Since our experimental approach uses catalytically dead dCas9 lacking cleavage activity, sgRNA activities are unlikely to explain segmental changes in gene expression. In addition, the sgRNAs are not enriched in the segmental deletion regions, which further excludes the possibility that CRISPR perturbation leads to segmental deletion (Supp. Fig. 8). Separately, we also applied recent computational approaches that have been developed to identify clones from single-cell transcriptomes alone [18, 19]. However, we could not identify clones, likely because our dataset does not satisfy all the criteria of these approaches.

Since major clones have the most number of cells, they are the most proliferative. To understand why the major clones expand more than other cells, we overlapped their genomic features with known oncogenes and tumor suppressors. We found that amplified regions in major Clone 0 overlap with oncogenes including MYC, while deleted regions overlap with tumor suppressor genes including TP53 and BRCA1 (Fig. 2F). We observe similar results in other clones (Supp. Fig. 9).

Finally, to determine the optimal experimental parameters for sgRNA-based clonal analysis from single cell experiments, we performed power analysis (Fig. 2G, Supp. Fig. 10). Assuming an sgRNA overlap rate of 75% in clonal cells (Fig. 1E-F), we calculated *p*-values as a function of sgRNA library size and the number of sgRNAs detected per cell. Overall, it is easier to identify clones with larger sgRNA libraries and more sgRNAs per cell. As a rule of thumb, a library size of at least 1000 sgRNAs with at least 10 sgRNAs per cell is sufficient for clonal analysis, which were satisfied in published studies [4, 5].

## Conclusions

In summary, this study highlights the importance of clone identification in single-cell CRISPR screens. We developed a computational pipeline to identify clonal cells without genomic DNA sequencing or additional engineering of clonal barcodes. We found that clonal expansion in single-cell CRISPR screens contributes to bias and leads to false discovery. Thus, experimental conditions that reduce clonal expansion or computational filtering of clonal cells will improve the reliability of single-cell CRISPR screens. This analysis could also be useful for understanding how distinct clones respond to perturbation.

## Methods

### Experimental details

#### Cell culture

MDA-MB-231 cells were purchased from ATCC and cultured with alpha modified MEM medium (Sigma) with supplement of 10% FBS, 1 mM sodium pyruvate (Gibco), 10 mM HEPES (Sigma), 1X Glutamax supplement (Thermo Fisher), 1X MEM non-essential amino acid (Sigma), 1 mg/mL insulin, 1 ng/mL hydrocortisone, 25 μg/mL EGF and pen/strep at 37C and 5% CO2 [20]. Lenti-X 293 T cells are purchased from Takara and cultured with DMEM medium with 10% of FBS. Cells were checked monthly for mycoplasma. To repress the activity of regulatory elements, we utilized catalytic dead Cas9 (dCas9) fused with the KRAB repressor domain. This effector lacks the ability to cut genomic DNA. To generate the stably expressed dCas9-KRAB, we used lenti-dCas9-KRAB-blast (Addgene ID: 89567) to package into lentivirus and infected MDA-MB-231 cells.

#### sgRNA library construction

sgRNA library construction was performed as described in Xie et al., 2019 [4]. Briefly, 20,139 single stranded sgRNA were synthesized by Genescript, and amplified by NEBNext High-Fidelity PCR master mix to make them double stranded. sgRNAs were inserted into the BsmBI-digested CROPseq-Guide-Puro backbone (Addgene ID: 86708) with Gibson Assembly. The final plasmid library was amplified and purified through electroporation under ampicillin selection overnight. ZymoPURE plasmid maxiprep kit was used to extract the plasmid library.

#### Virus packaging and infection

Virus packaging and infection was performed as described in Xie et al., 2019 [4]. Briefly, the sgRNA plasmid library was packaged into lentivirus by co-transfecting with pMD2.G and psPAX2 (Addgene ID 12259 and 12,260) to Lenti-X 293 T cells using linear polyethylenimine (Transporter 5® Transfection Reagent, Polysciences). Medium was changed after 8 h of transfection, and the supernatant was collected and filtered through a 0.22 um filter 48 h after transfection. Virus was concentrated with the Lenti-X concentrator (Clontech) according to the manufacturer’s instructions.

We performed lentivirus infection of MDA-MB-231 cells in 24-well plate format. To maximize MOI, we infected MDA-MB-231 with a serial dilution of virus, and focused sequencing efforts on healthy cells with the highest MOI, as in [5]. In this experiment, 4 wells of cells under antibiotic selection for 24 days were used. We confirmed the per-cell titer of sgRNAs by performing single-cell RNA seq.

#### Single-cell CRISPR screen library construction

Single-cell library construction was performed as described in Xie et al., 2019 [4]. Briefly, cells were labeled with 10 different antibodies using the cell hashing protocol [13]. Twenty-five thousand cells were loaded into each of 6 lanes of the 10X Chromium Single-Cell 3′ V3 RNA-seq kit. Transcriptome libraries were constructed following the manufacturer’s instructions. sgRNA libraries were amplified from 50 ng of transcriptome PCR1 products using the SI primer and sgRNA enrichment primer. Nextera indexes were added in the second run of PCR. We used 1.6X of SPRI purification to clean up the final sgRNA libraries (~ 500 bp).

#### Sequencing

Libraries were sequenced on the Illumina NextSeq (R1 28bp, R2 56bp, and idx1 8 bp) and the Illumina NovaSeq (R1 150bp, R2 150bp, idx1 8 bp). Each transcriptome library has ~ 300 M reads, and each sgRNA library and Cell Hashing library has ~ 30 M reads.

### Computational analysis

#### sgRNA library design

We combined 4451 breast cancer GWAS SNPs [21] into 133 1-Mb loci, which are across all 23 chromosomes. We identified regulatory elements based on ATAC-seq [22] and H3K27ac ChIP-seq signals [20]. We called the ATAC-seq signals using the function callpeak from macs2, and we quantified the H3K27ac ChIP-seq signals within the ATAC-seq peaks by using ‘featureCounts’. The cutoff for H3K27ac ChIP-seq signals is more than 1 RPKM. There are 884 promoters and 1214 enhancers after all the filterings. To find the sgRNAs targeting the enhancers, we expanded the ATAC-seq signal peak to 500 bp, and identified 10 non-overlapping sgRNAs spanning each targeted region. We aligned the sgRNAs in the human genome with BLAST to reduce off targets. Overall, 99.9% of the sgRNAs in the library are aligned to only one region of the human genome (Supp. Fig. 1).

#### Data preprocessing and hits calling

Data processing was performed as described in [4]. Briefly, transcriptome libraries were mapped to the human reference genome (hg38) with Cellranger software (version 3.1), with expected cell number of 20,000 and default parameters. Cell hashing libraries were mapped with the cellranger software together with the transcriptome libraries. We searched all the possible sgRNA sequences in fastq files with regular expression analysis, allowing one base pair of mismatch.

Experimental doublets were removed through cell hashing [14]. We performed Louvain clustering [23], and we removed one distinct cluster of cells that highly expressed mitochondrial genes (Supp. Fig. 1). sgRNA counts were summarized for each cell, and UMIs were processed with the ‘directional’ method described in UMI-tools [24]. For each cell, we filter out low UMI sgRNA by applying a saturation curve method as described in Drop-seq [25]. The metrics for all the libraries can be found in Supplementary Table 1. Overall, the average UMI for each detected sgRNA in each cell is 15.1.

We grouped all the sgRNAs for each perturbed region and calculated if the expression level of each gene changed significantly due to perturbation using hypergeometric test [4]. To adjust for false discovery, we calculated the background *p*-value for each gene by performing the hypergeometric test with 10,000 randomization of sgRNA combinations [4]. The Significance Score for each gene is then the observed log_10_p minus the randomized background log_10_p.

#### Grouping cells into clones using sgRNA information

The input is the sgRNA matrix with cell barcodes as columns and all the sgRNA sequences as rows. To statistically test whether two cells belong to the same clone, we use multiplexed sgRNAs as clonal barcodes and we use the hypergeometric test (‘scipy.stats.hypergeom.sf’ function) with the parameters below:

- x: The number of overlap sgRNA between the two cells minus one.
- M: The size of the sgRNA library.
- n: The number of sgRNA in cell 1.
- N: The number of sgRNA in cell 2.

The agglomerative algorithm to group cells into clones proceeds as follows. Clonal information is stored as a Python dictionary data structure D, initially empty. We iterate through each cell c in the population C and compare sgRNA overlap to cells in D. If cell c does not have significant sgRNA overlap with any cell in D, then c is added to D as a new clone. If cell c has significant sgRNA overlap with exactly 1 clone d in D, then c is added to clone d. The Bonferroni-corrected *p*-value cutoff for the same clone is 0.05. Cells with statistically significant sgRNA overlap with multiple distinct clones were removed from downstream analysis because they are likely cell doublets. The pseudocode is below:

- D = empty clone dictionary

for each cell c in the set of all cells C:

- if c has sgRNA overlap with 0 clones in D, then add c as a new clone to D
- if c has sgRNA overlap with exactly 1 clone d in D, then add c to clone d
- if c has sgRNA overlap with > 1 clones in D, then mark c as doublet

#### Simulations of clones to assess method robustness

To estimate the robustness of our approach, we simulated 1000 clonal cells, each having 30 sgRNAs where 75% of the sgRNAs were derived from a clone and the remaining 25% of the sgRNAs were randomly selected from the full sgRNA library. Next, we simulated 1000 non-clonal cells by randomly selecting 30 sgRNAs from the whole sgRNA library. To assess robustness, we then repeated our analysis with the 2000 simulated cells (Supp. Fig. 2).

#### Visualizing segmental copy number changes from gene expression

We normalized the transcriptome count matrix with the sequencing depth for each cell by calculating counts per millions (cpm). We filtered out genes expressed in less than 10% of all cells and calculated the normalized expression z-score of cpm matrix for each expressed gene relative to all sequenced cells with the function stats.zscore(cpm_matrix, axis = 1, ddof = 1). We then arranged the expressed genes based on the chromosome position and plotted the heatmap with the ‘imshow’ function of the matplotlib package. More details can be found in the jupyter notebook here: https://github.com/yihan1119/Group_clone/blob/main/Notebooks/All_clones_zscore-Github.ipynb.

## Supplementary Information

**Additional file 1: Supplementary Figure 1.** Quality control of libraries. **Supplementary Figure 2.** Pipeline performance and accuracy test. **Supplementary Figure 3.** Single cell sgRNA libray UMI cutoff test. **Supplementary Figure 4.** Detection of clonal cells in publicly available datasets. **Supplementary Figure 5.** Clonal cells increase the noise. **Supplementary Figure 6.** Non-clonal cells do not share the genomic features of clonal cells. **Supplementary Figure 7.** Clonal cells share similar transcriptomes. **Supplementary Figure 8.** Segmental deletions are not caused by CRISPR perturbation. **Supplementary Figure 9.** Copy number changes overlap with oncogenes/ tumor suppressors in major clones. **Supplementary Figure 10.** Power analysis for different sgRNA overlap rate.

**Additional file 2.**

## Data Availability

The sequencing data generated in this study have been deposited to the Gene Expression Omnibus (GEO) under the accession (GSE185995, https://www.ncbi.nlm.nih.gov/geo/query/acc.cgi?acc=GSE185995) We have deposited example scripts to Github (https://github.com/yihan1119/Group_clone).

## Abbreviations

CRISPR: Cclustered regularly interspaced short palindromic repeats
sgRNA: Cingle guide RNA
MOI: Multiplicity of infection
