# Supplementary material for: Computational identification of clonal cells in single-cell CRISPR screens
Source: BMC Genomics. 2022 Feb 15;23:135. doi: 10.1186/s12864-022-08359-1 (PMC8845350; doi:10.1186/s12864-022-08359-1)
Supplement: Supplementary file 1 — Additional file 1: Supplementary Figure 1. Quality control of libraries. Supplementary Figure 2. Pipeline performance and accuracy test. Supplementary Figure 3. Single cell sgRNA libray UMI cutoff test. Supplementary Figure 4. Detection of clonal cells in publicly available datasets. Supplementary Figure 5. Clonal cells increase the noise. Supplementary Figure 6. Non-clonal cells do not share the genomic features of clonal cells. Supplementary Figure 7. Clonal cells share similar transcriptomes. Supplementary Figure 8. Segmental deletions are not caused by CRISPR perturbation. Supplementary Figure 9. Copy number changes overlap with oncogenes/ tumor suppressors in major clones. Supplementary Figure 10. Power analysis for different sgRNA overlap rate. [file 12864_2022_8359_MOESM1_ESM.pdf]

Supplementary Figure 1: Quality control of libraries

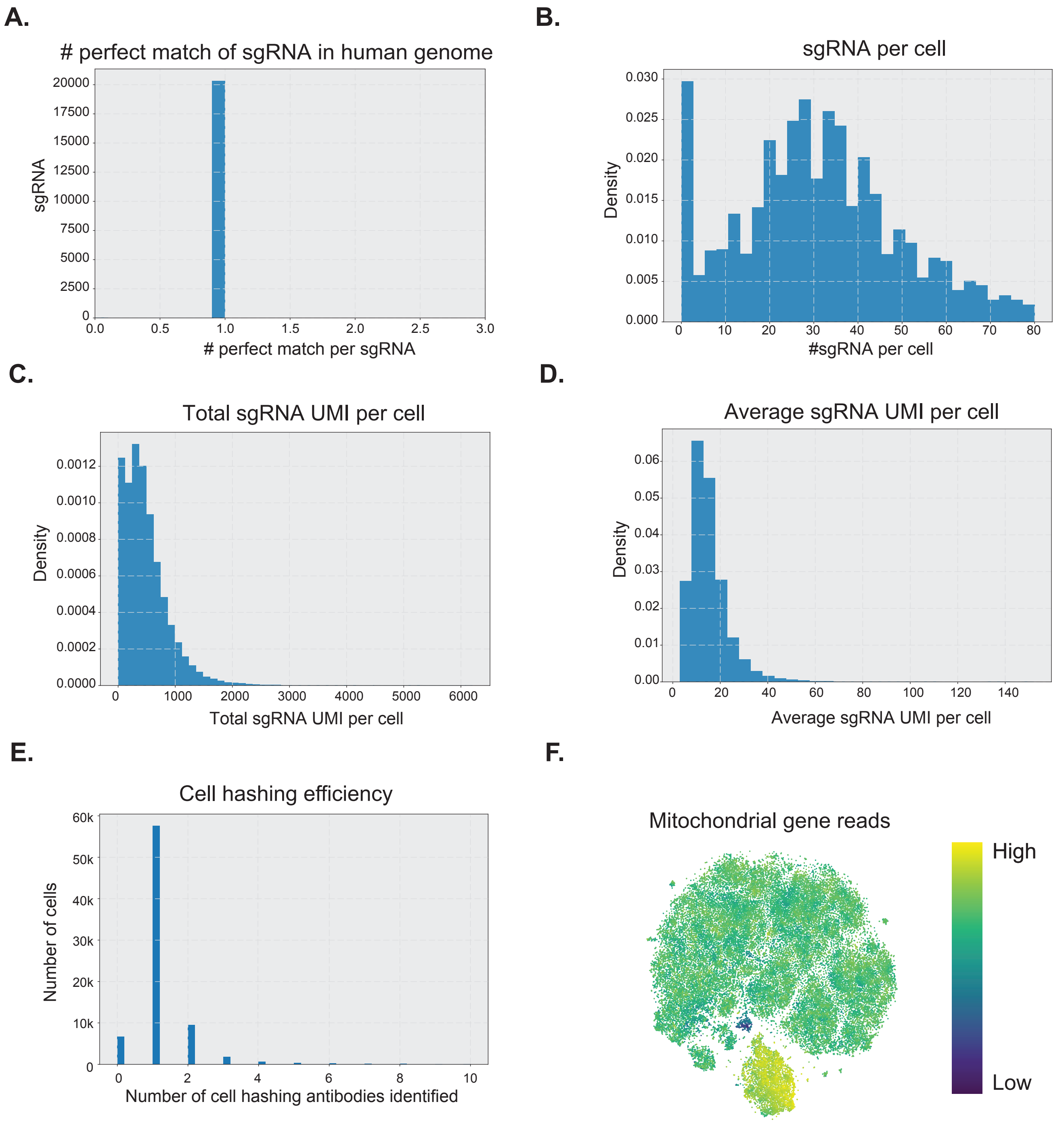

Supplementary Figure 1: Quality control of libraries

A. The histogram of number of perfect match sites between sgRNA and human genome. We used BLAST to evaluate the sgRNA specificity, and 99.9% of the sgRNAs only have one perfect match site in the human genome.

B. The distribution of the number of sgRNAs identified per cell. High MOI infection leads to high number of sgRNA per cell.

C. The histogram of total UMI counts for sgRNAs in each cell. On average, the total number of UMIs detected for sgRNAs in each cell is 483.6.

D. The histogram of UMI counts for individual sgRNAs in each cell. On average, the UMI of each sgRNA in each cell is 15.1.

E. The bar chart of cell hashing efficiency. 75% of cells have exactly one cell hashing antibody identified.

F. This t-SNE plot identifies a cluster of cells with high mitochondrial gene content.

Supplementary Figure 2: Pipeline performance and accuracy test

A.

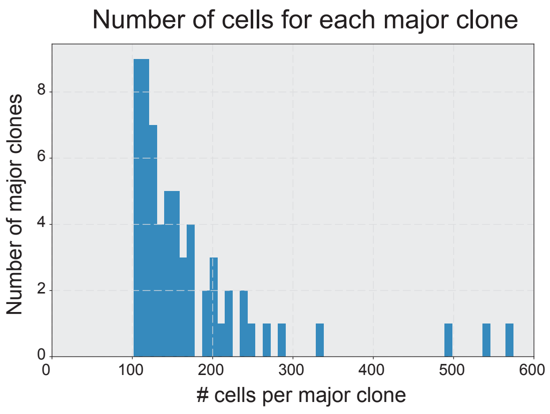

B.

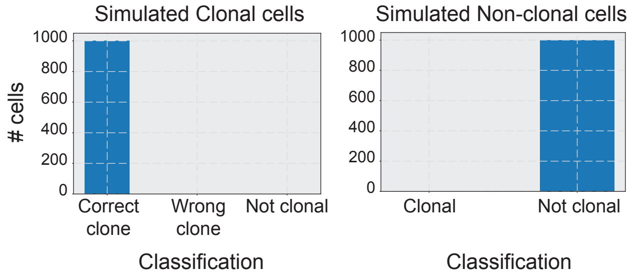

C.

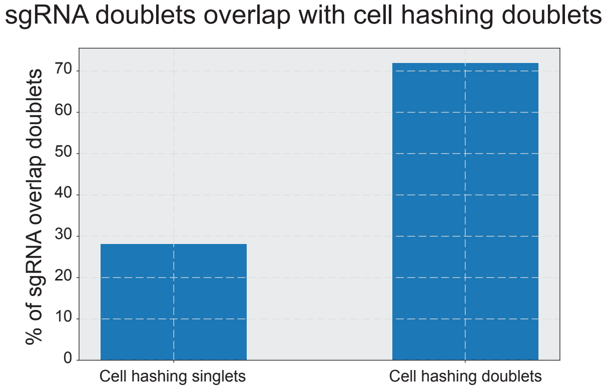

Supplementary Figure 2: Pipeline performance and accuracy test.
A. Distribution of the number of cells per major clone. The major clone is defined as clones with more than 100 cells, and there are 54 major clones.
B. We simulated clonal and non-clonal cells to assess the accuracy of the pipeline. We randomly generated 1000 clonal cells (left) and 1000 non-clonal cells (right). Next, we applied our computational approach to group simulated cells into clones. The histograms show that all the simulated clonal cells were correctly called as clonal and in the correct clone group. In addition, all simulated non-clonal cells were correctly called as non-clonal.
C. Here, we test the hypothesis that sgRNA overlap can be used as an indicator of cell doublets. If this is the case, then we expect cell doublets identified by the established cell hashing method (which we will refer to as “cell hashing doublets”) to have increased likelihood to have significant sgRNA overlap with multiple clones, relative to cell hashing singlets. Thus, we repeated our analysis to identify sgRNA doublets (cells that have significant sgRNA overlap with multiple clones), but this time using all cells (cell hashing singlets and doublets). We observed that 72% of sgRNA doublets are cell hashing doublets. Given that only 25% of cells are cell hashing doublets, this 10-fold enrichment is significant.

Supplementary Figure 3: single cell sgRNA libray UMI cutoff test

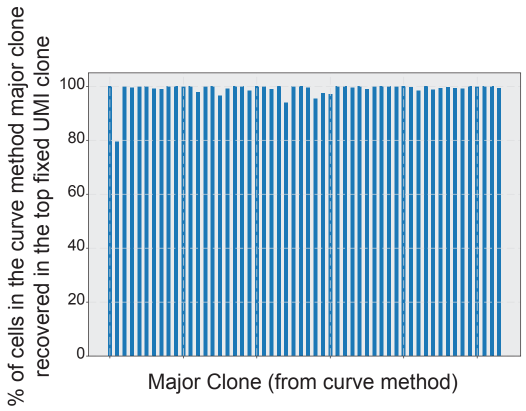

Supplementary Figure 3: Single cell sgRNA library UMI cutoff test

To confidently identify the sgRNAs in each cell, our standard procedure uses the ‘curve method’ described in Drop-seq paper. This approach dynamically identifies a UMI cutoff to identify sgRNAs. This approach filters out the noise of low UMI sgRNAs. As a result, the the average UMI cutoff for sgRNAs is 4.5.

Here, to test an alternative approach to identifying sgRNAs in each cell, we applied a fixed UMI cutoff of 2 UMI for all the cells. We found that this simple approach recovers all of the major clones identified from the curve method (above). Lowering the UMI cutoff results in 68 major clones instead of 54.

Supplementary Figure 4: Detection of clonal cells in publicly available datasets.

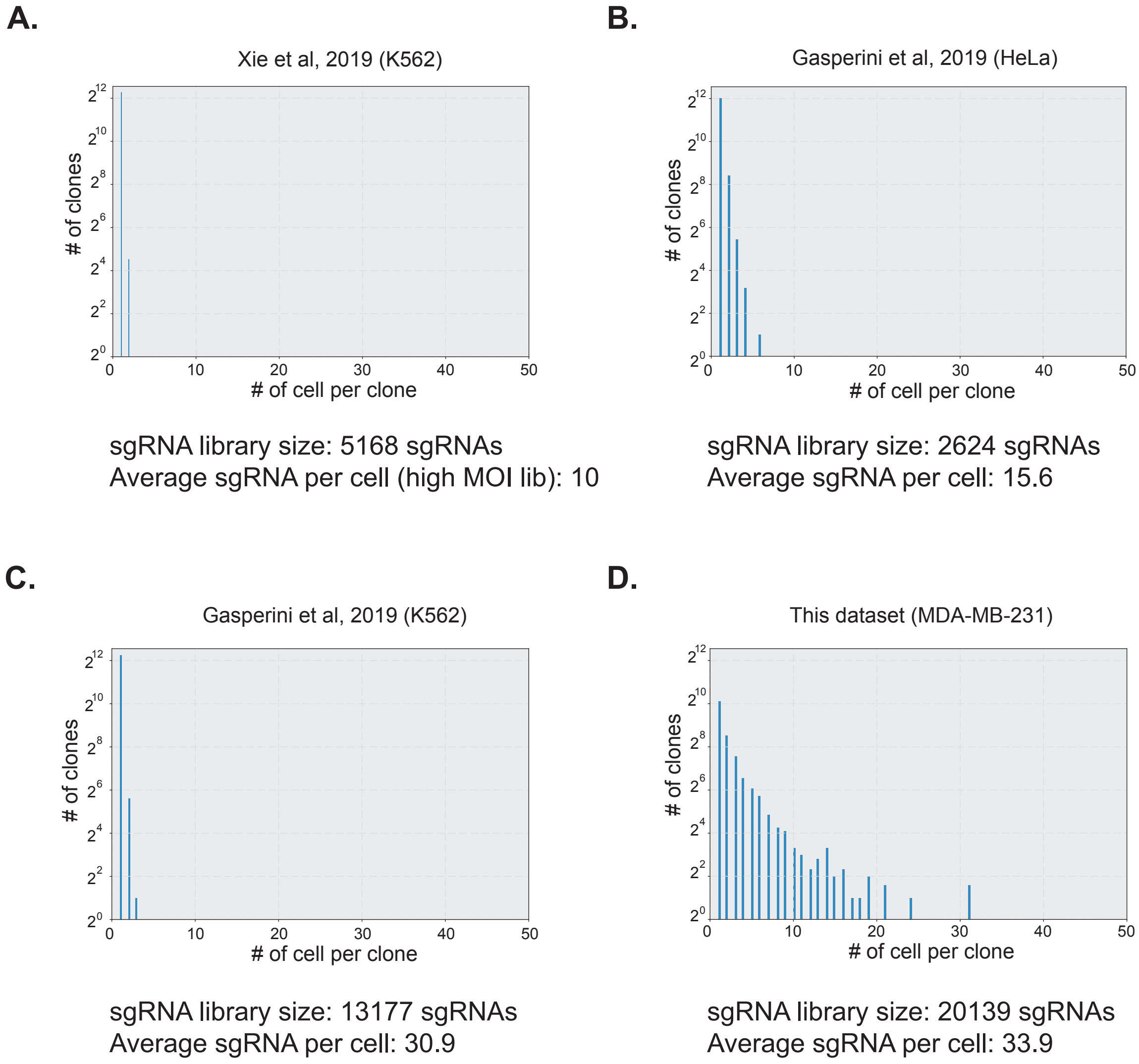

Supplementary Figure 4: Detection of clonal cells in publicly available datasets.

A. We randomly selected 5000 cells from each dataset to estimate clonality. This plot shots the distribution of clone sizes (the number of cells per clone). In Xie et al, we estimate that 99% of cells are non-clonal.

B. Distribution of clone sizes (the number of cells per clone) in Gasperini et al (HeLa). We estimate that 83% of cells are non-clonal.

C. Distribution of clone sizes (the number of cells per clone) in Gasperini et al (K562). We estimate that 98% of cells are non-clonal.

D. Distribution of clone sizes (the number of cells per clone) in this paper. We estimate that 22% of MDA-MB-231 cells are non-clonal.

Supplementary Figure 5: Clonal cells increase the noise.

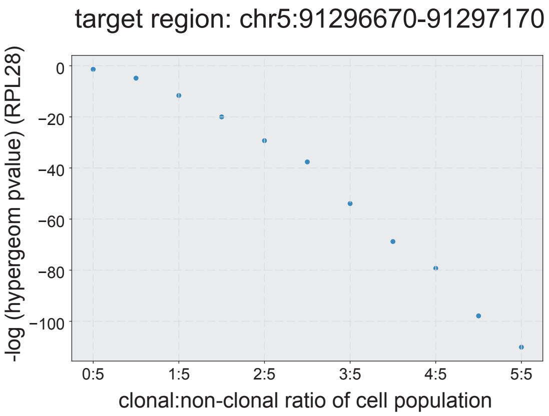

Supplementary Figure 5: Clonal cells increase the noise.
The p-value of RPL28 change as more clonal cells are added in the analysis of differential expression analysis of chr5:91296670-91297170. Clonal cells make up from 0% (left) to 50% (right) of the total population of cells. The non-clonal population is fixed.

Supplementary Figure 6: Non-clonal cells do not share the genomic features of clonal cells.

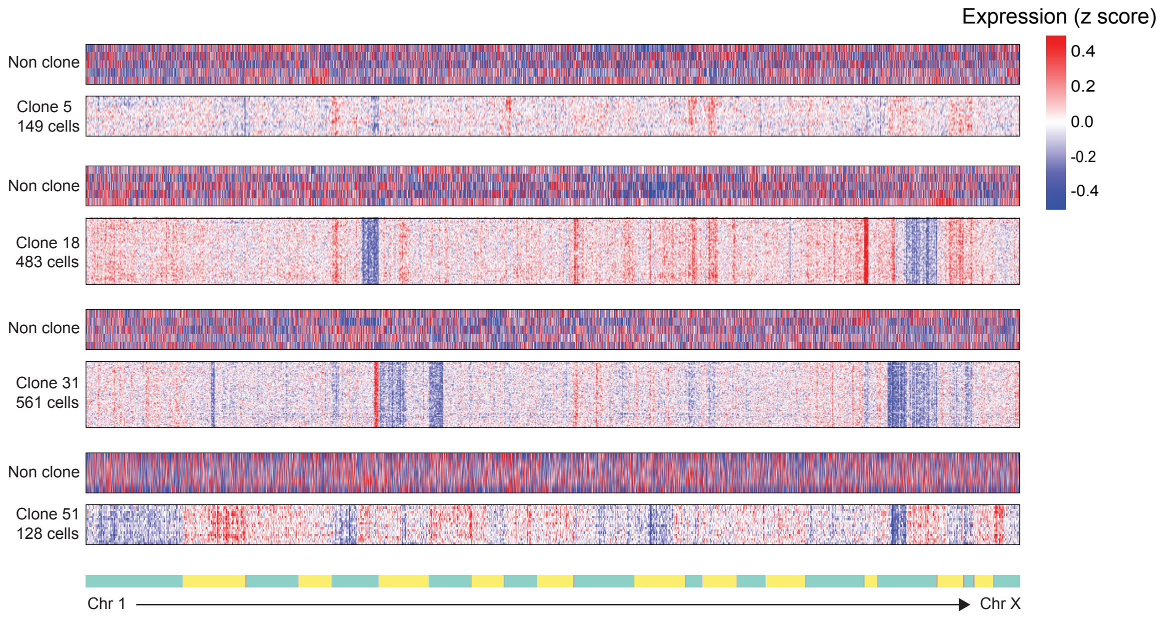

Supplementary Figure 6: Non-clonal cells do not share the genomic features of clonal cells.
For each clone, we identified the top 5 non-clonal cells using a Chi-square test. The heatmaps show the z-score normalized expression of all genes ordered by chromosomal coordinate, for (top) top-ranked non-clonal cells and (bottom) clonal cells. Examples for 4 major clones are shown.

Supplementary Figure 7: Clonal cells share similar transcriptomes.

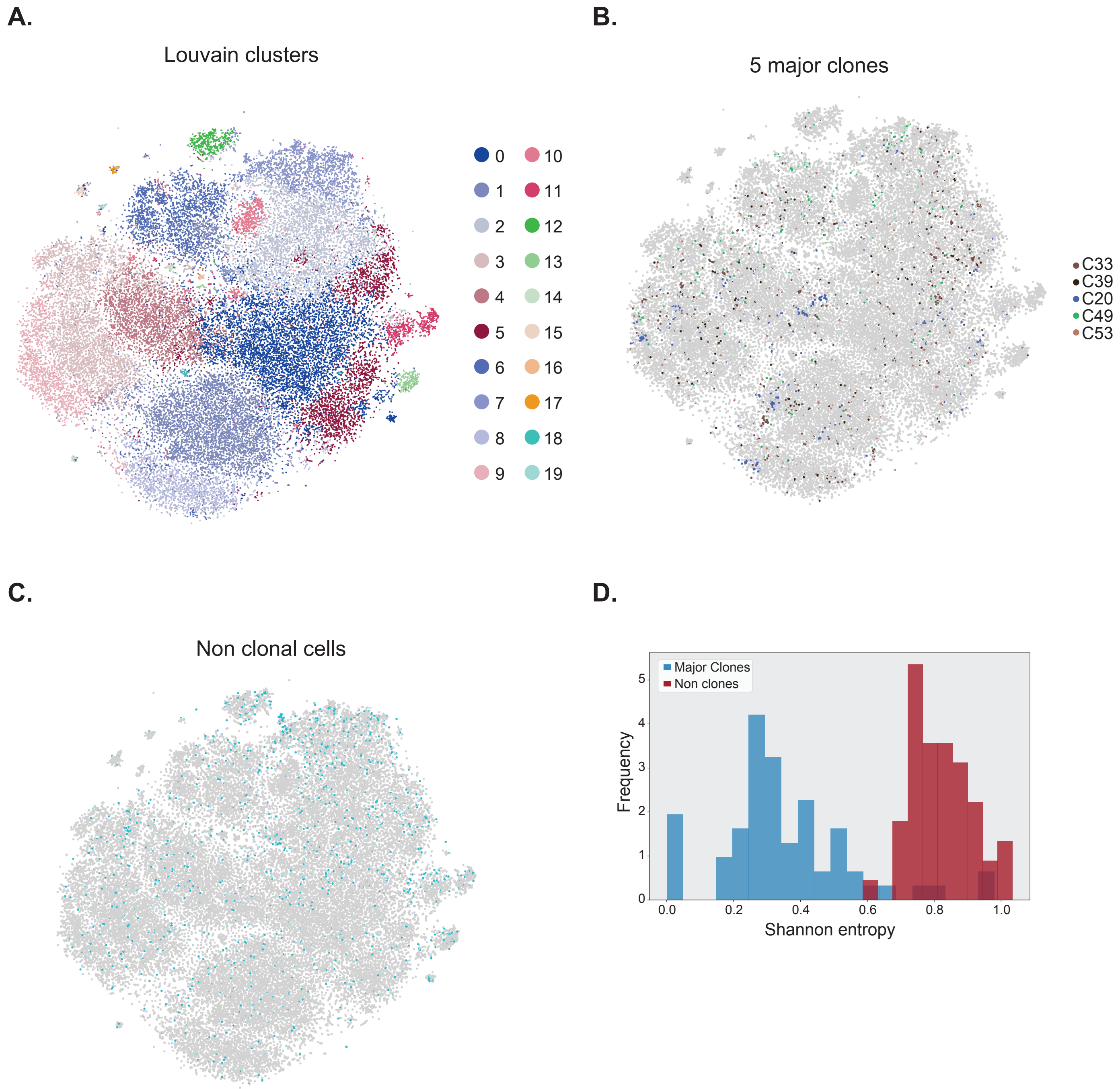

Supplementary Fig. 7: Clonal cells share similar transcriptomes.
A. Shown is a t-SNE plot with cells colored by Louvain cluster.
B. Feature plot of 5 major clones.
C. Feature plot of non-clonal cells.
D. The distribution of Shannon entropy for major clones is shown in blue. Here, we define Shannon entropy based on Louvain clusters. To simulate the entropy of non-clones (red), we randomly sampled non-clonal cells where the number of cells sampled is equal to the number of cells in major clones.

Supplementary Figure 8: Segmental deletions are not caused by CRISPR perturbation

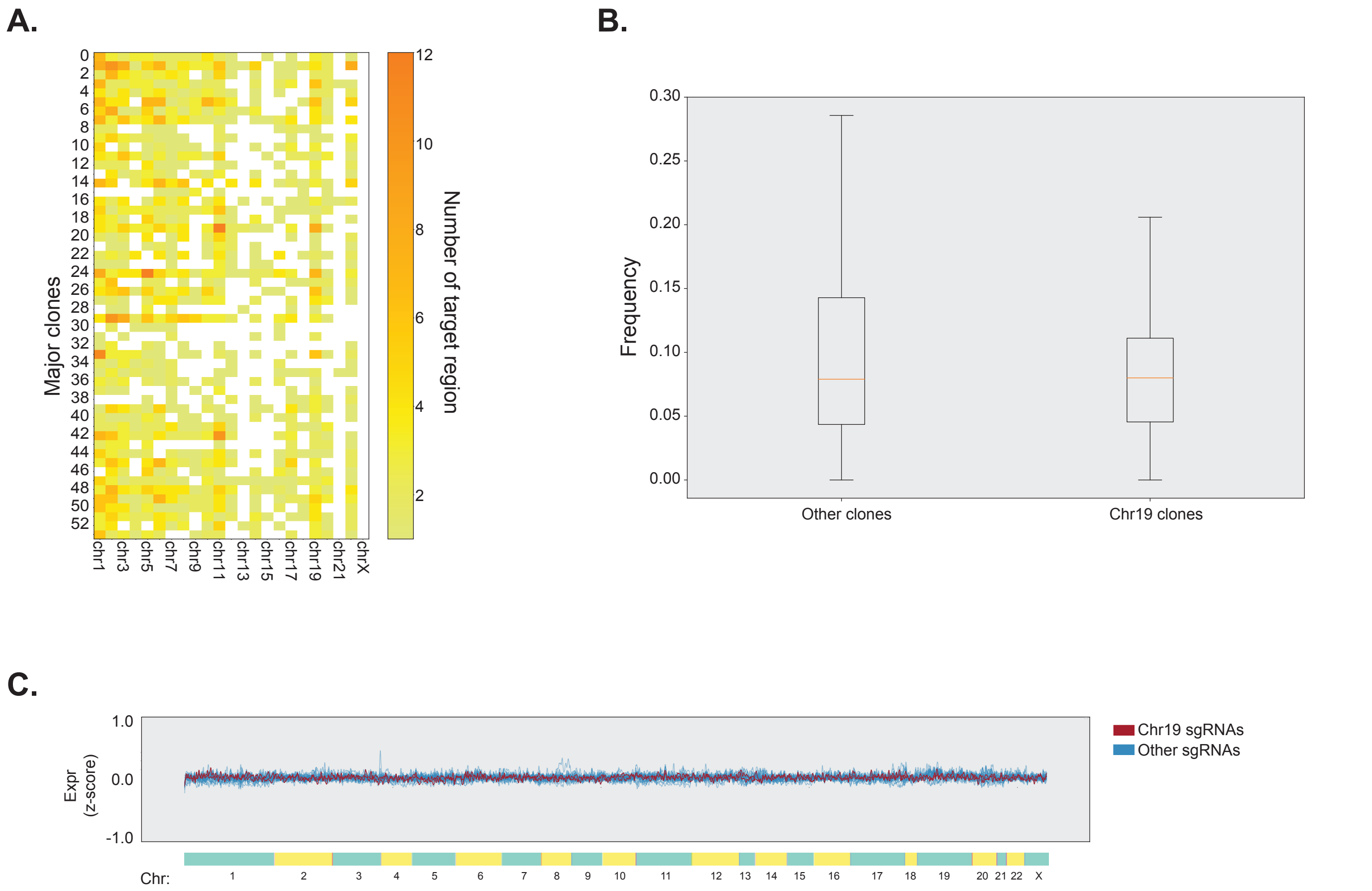

Supplementary Figure 8: Segmental deletions are not caused by CRISPR perturbation.

A. The number of target regulatory elements in each major clone. The enriched regions are based on the sgRNAs in more than 10% of the major clone cells.

B. The proportion of sgRNAs targeting chr19 enhancers in: major clones with chr19 deletion and other major clones without chromosome 19 deletion. There are 3625 clonal cells with chr19 deletion, and 5745 major clonal cells without chr19 deletion.

C. To test if sgRNA off-targeting could cause segmental changes in gene expression, we focused major clone 18, which contains 27 sgRNAs. We identified the cells expression each of these sgRNAs and removed those in clone 18. We then examined plotted the expression z-score trace of the remaining cells. The results show that non-clonal cells expressing any of the 27 sgRNAs do not have segmental deletion of chr19.

Supplementary Fig. 9: Copy number changes overlap with oncogenes/ tumor suppressors in major clones.

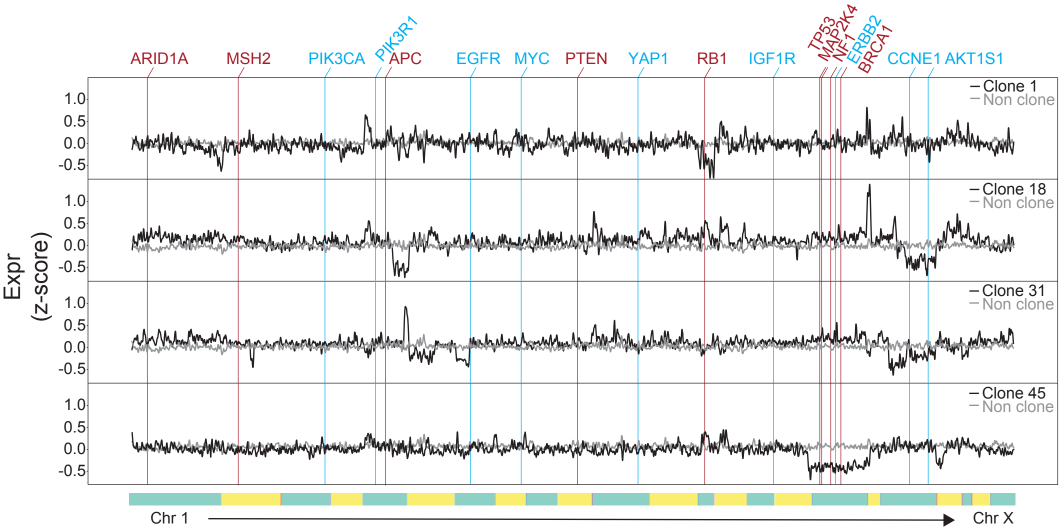

Supplementary Fig. 9: Copy number changes overlap with oncogenes/ tumor suppressors in major clones. For several clones, shown is the average z-score normalized expression of genes, ordered by chromosomal coordinate. Several tumor suppressors (red) and oncogenes (cyan) that overlap potential regions of segmental amplification or deletion, respectively, are labeled.

Supplementary Figure 10: Power analysis for different sgRNA overlap rate.

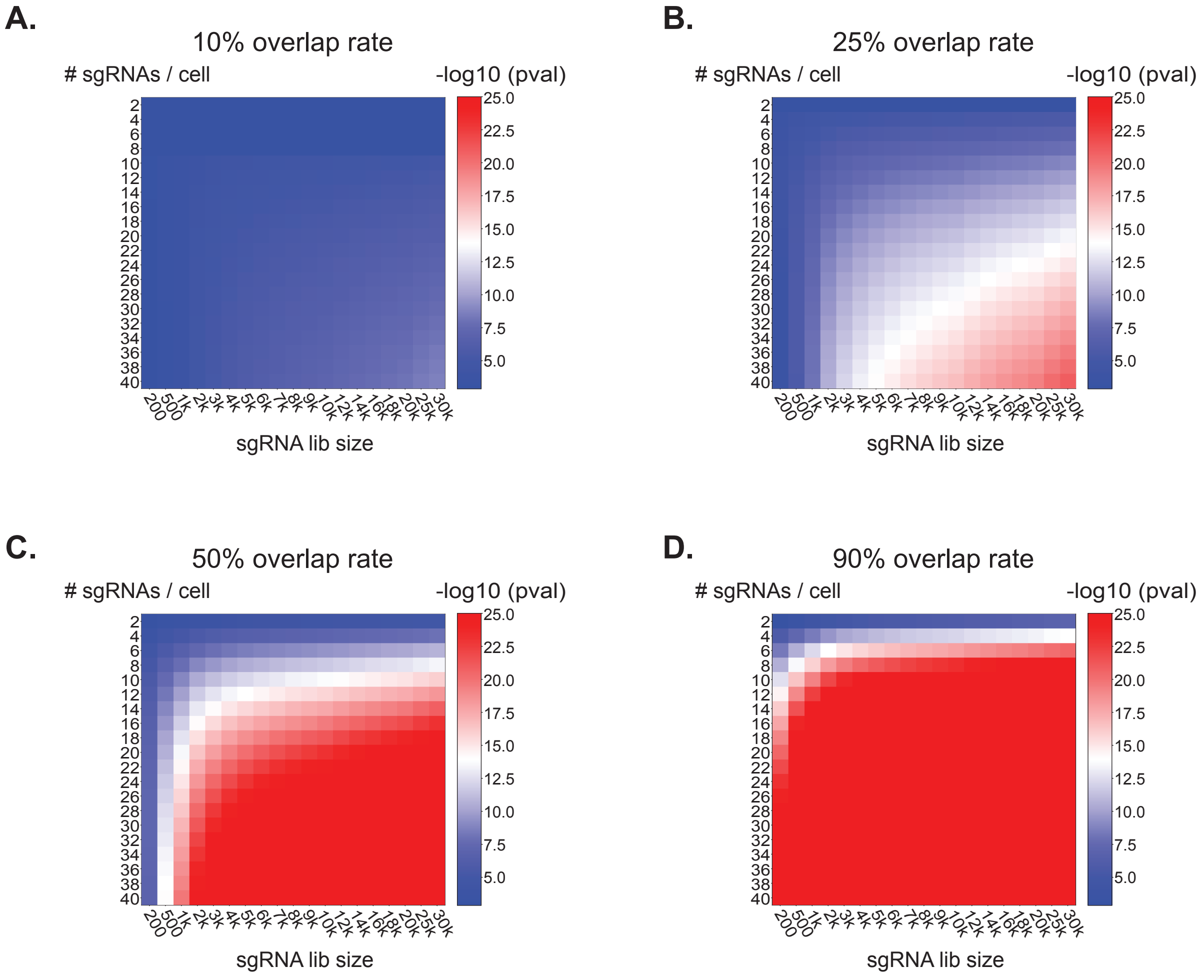

Supplementary Figure 10: Power analysis for different sgRNA overlap rate
A. As in Figure 2G, but for 10% of sgRNA overlap rate between two cells.
B. As in Figure 2G, but for 25% of sgRNA overlap rate between two cells.
C. As in Figure 2G, but for 50% of sgRNA overlap rate between two cells.
D. As in Figure 2G, but for 90% of sgRNA overlap rate between two cells.
